# *Trichoderma virens* β-glucosidase I (*BGL*I) gene; expression in *Saccharomyces cerevisiae* including docking and molecular dynamics studies

**DOI:** 10.1186/s12866-017-1049-8

**Published:** 2017-06-21

**Authors:** Gammadde Hewa Ishan Maduka Wickramasinghe, Pilimathalawe Panditharathna Attanayake Mudiyanselage Samith Indika Rathnayake, Naduviladath Vishvanath Chandrasekharan, Mahindagoda Siril Samantha Weerasinghe, Ravindra Lakshman Chundananda Wijesundera, Wijepurage Sandhya Sulochana Wijesundera

**Affiliations:** 1 Department of Chemistry, Faculty of Science, University of Colombo, Colombo, Sri Lanka; 2 Department of Plant Sciences, Faculty of Science, University of Colombo, Colombo, Sri Lanka; 3 Department of Biochemistry and Molecular Biology, Faculty of Medicine, University of Colombo, Kynsey Road, Colombo, 08 Sri Lanka

**Keywords:** Lignocellulose, β-glucosidase, Recombinant *S.cerevisiae*, Molecular docking, Molecular dynamics simulations, Homology modeling, Simultaneous saccharification and fermentation

## Abstract

**Background:**

Cellulose, a linear polymer of β 1–4, linked glucose, is the most abundant renewable fraction of plant biomass (lignocellulose). It is synergistically converted to glucose by endoglucanase (EG) cellobiohydrolase (CBH) and β-glucosidase (BGL) of the cellulase complex. BGL plays a major role in the conversion of randomly cleaved cellooligosaccharides into glucose. As it is well known, *Saccharomyces cerevisiae* can efficiently convert glucose into ethanol under anaerobic conditions. Therefore, *S.cerevisiae* was genetically modified with the objective of heterologous extracellular expression of the *BGL*I gene of *Trichoderma virens* making it capable of utilizing cellobiose to produce ethanol.

**Results:**

The cDNA and a genomic sequence of the *BGLI* gene of *Trichoderma virens* was cloned in the yeast expression vector pGAPZα and separately transformed to *Saccharomyces cerevisiae*. The size of the *BGL*I cDNA clone was 1363 bp and the genomic DNA clone contained an additional 76 bp single intron following the first exon. The gene was 90% similar to the DNA sequence and 99% similar to the deduced amino acid sequence of 1,4-β-D-glucosidase of *T. atroviride* (AC237343.1). The BGLI activity expressed by the recombinant genomic clone was 3.4 times greater (1.7 x 10^−3^ IU ml^−1^) than that observed for the cDNA clone (5 x 10^−4^ IU ml^−1^). Furthermore, the activity was similar to the activity of locally isolated *Trichoderma virens* (1.5 x 10^−3^ IU ml^−1^). The estimated size of the protein was 52 kDA. In fermentation studies, the maximum ethanol production by the genomic and the cDNA clones were 0.36 g and 0.06 g /g of cellobiose respectively. Molecular docking results indicated that the bare protein and cellobiose-protein complex behave in a similar manner with considerable stability in aqueous medium. The deduced binding site and the binding affinity of the constructed homology model appeared to be reasonable. Moreover, it was identified that the five hydrogen bonds formed between the amino acid residues of BGLI and cellobiose are mainly involved in the integrity of enzyme-substrate association.

**Conclusions:**

The BGLI activity was remarkably higher in the genomic DNA clone compared to the cDNA clone. Cellobiose was successfully fermented into ethanol by the recombinant *S.cerevisiae* genomic DNA clone. It has the potential to be used in the industrial production of ethanol as it is capable of simultaneous saccharification and fermentation of cellobiose. Homology modeling, docking studies and molecular dynamics simulation studies will provide a realistic model for further studies in the modification of active site residues which could be followed by mutation studies to improve the catalytic action of BGLI.

## Background

The conversion of cellulose and hemicellulose of lignocellulosic biomass into ethanol is a promising solution to the anticipated future fuel crisis. The sugar monomers of these two major components of plant biomass can be fermented to ethanol [1]. Therefore, second generation biofuel production based on enzymatic conversion of cellulosic biomass into ethanol was selected as a key area in the development of renewable energy technology in the 1980s [2, 3].

Cellulose consists mainly of long polymers of β 1–4, linked glucose units [4]. Cellulase is an enzyme complex consisting of endoglucanase (endo-1,4-β-D-glucanase, EGL, EC 3.2.1.4); cellobiohydrolase or exoglucanase (exo-1,4-β-D-glucanase, CBH, EC 3.2.1.91) and β-glucosidase (1,4-β-D-glucosidase, BGL, EC 3.2.1.21) that act synergistically to convert cellulose to glucose [5, 6]. The accumulation of cellooligosaccharides (cellobiose and cellotriose) inhibits the function of both CBH and EGL enzymes in simultaneous saccharification and therefore BGL plays a major role in the efficient conversion of the randomly cleaved inhibitory form of cellooligosaccharides into utilizable non-inhibitory glucose units [7].

Cellulases are produced by a variety of microorganisms including filamentous fungi and bacteria [8, 9]. Filamentous fungi are naturally excellent protein secretors and produce industrially important enzymes in feasible amounts [10]. Therefore, much attention has been given to fungal cellulolytic systems over those of bacteria [11]. It is reported that fungal strains secrete higher amounts of cellulases than bacterial species. The fungal species *Trichoderma* has been studied extensively as it is known to produce higher amounts of cellulases compared to other fungal species [12–14].

*Saccharomyces cerevisiae* can efficiently convert glucose into ethanol under anaerobic conditions [15]. It has the ability to propagate rapidly by budding and fission. This inherent phenomenon can be employed as a conclusive tool for successive utilization and subsequent fermentation of lignocelluloses into ethanol by expressing cellulase in *Saccharomyces cerevisiae* [16]. BGL has been expressed in *Saccharomyces cerevisiae* by many researchers with the objective of producing ethanol from lignocellulosic biomass [17, 18]. However, in many previous studies the expression levels have been reported to be considerably lower than the native host species, probably due to incompatibility of the signal peptide and the promoter of the yeast expression system [17, 19, 20]. The present study describes the characterization, cloning and expression of both genomic and cDNA clones of *BGL*I in *S. cerevisiae* from locally isolated *Trichoderma virens* using yeast integrative vector pGAPZα. The vector consists of the α-mating factor (MFα) signal sequence with the glyceraldehyde 3-phosphate dehydrogenase (GAP) promoter driven expression system [21–23]. The expression of both genomic and cDNA clones of *BGL*I by recombinant *S.cerevisiae* was compared with the objective of analyzing the effect of introns on expression in the eukaryotic system as the presence of introns have been known to enhance expression. Absence of a three-dimensional (3D) structure is a limitation in understanding the structural features and properties of BGLI. Therefore, a 3D structure of BGLI was built using homology modeling with quality assessments. Molecular dynamics (MD) simulations and protein docking studies were carried out to investigate the docking of substrates to the catalytic site of the enzyme and to study how enzyme dynamics are affected by cellulose. MD simulation is one of the most important tools for the computational study of bio-molecules. It provides the time-dependent behavior of the bio-molecule as well as comprehensive information of the conformational changes in the molecular system [24].

## Methods

### β-glucosidase (BGLI) activity assay of *Trichoderma virens*

A phenotypically characterized, local *Trichoderma* isolate was subjected to PCR based internal transcribed spacer (ITS) analysis and confirmed as *Trichoderma virens*. The cultures for the *BGL*I enzyme assay was prepared by placing a 5 mm diameter mycelium disc removed from a 7 day old culture and inoculated into conical flasks (100 mL) containing 25 mL of Mandel’s medium (MM) [25] supplemented with 2% cellobiose as the sole carbon source [26]. Cultures were incubated at 30 °C, in a rotary shaker at 150 rpm and the enzyme extracts were harvested by filtration at 24 h, 48 h, 72 h, 96 h, 168 h, 192 h and 216 h intervals. Cell free enzyme extracts were obtained by centrifugation at 6200 *g* at 4 °C for 10 min and then freeze dried. A 200 μL volume of the enzyme extract was mixed with cellobiose 2.8 mL (15 mM) as the substrate in a citrate buffer (50 mM, pH 4.8). Thereafter, the reaction mixture was incubated at 50 °C in a water bath for 15 min followed by addition of 3 mL of 3,5-dinitrosalicyclic acid (DNS) solution into each reaction mixture and placed them in a boiling water bath for 15 min to develop the colour. The colour intensity was measured at 540 nm [27]. The enzyme activity was calculated with reference to the glucose standard graph and all experiments were performed in triplicate. One unit of BGL activity is defined as 1 μmol of glucose produced from cellobiose in 1 mL of enzyme volume per second (μmol ml^−1^ s^−1^) [28].

### Cloning of *BGL*I in yeast expression vector pGAPZα

Gene specific primers were designed with reference to the sequence homology and the open reading frame of *BGL*1 was identified using the nucleotide blast search and ORF finder in NCBI. (https://www.ncbi.nlm.nih.gov/orffinder/). Primers were designed with the objective of incorporating the α-mating factor signal sequence of pGAPZα vector (Invitrogen, USA) for extracellular expression of *BGL*1.

Genomic DNA was extracted from *T. virens* using a simple ﻿extraction method [29]. Genomic DNA was PCR amplified using *BGL*I gene specific primers containing restriction enzyme sites {BGLIFP: 5′-ATCGTGAATTCATGTTGCCCAAGGACT-3′(*Eco*RI) and BGLIRP: 5′-TTGATTCTAGATCAAGCTCTTTGCGCT-3′ (*Xba*I)}. The PCR cycling conditions were as follows; initial denaturation at 94 °C for 2 min followed by; 94 °C /30 s, 53 °C / 30 s, 72 °C/ 30 s, for 35 cycles and then at 72 °C / 7 min. The PCR product was electrophoresed on a 0.8% agarose gel and was observed under UV illumination.

To construct the cDNA of *BGL*I, RNA was extracted from fungal mycelium harvested on the sixth day of its highest BGL activity as described below. The mycelia were washed in phosphate buffer solution (1X PBS, pH 7.5) to remove pigments and other components in the medium. The RNA was extracted using the guanidium thiocyanate method [30] followed by DNase (Promega,USA) treatment. The concentration of extracted total RNA was determined and its purity was examined by agarose gel electrophoresis. RNA was reverse transcribed using MMLV (Promega,USA) and oligodT primer (Promega,USA). The synthesized cDNA was then subjected to PCR amplification using gene specific primers as mentioned above. Amplified *BGL*1 gene from both genomic DNA and cDNA of *T.virens* were separately purified and cloned into pGEM-T vector (Promega, USA). Thereafter, they were separately transformed into *E. coli* JM109 competent cells (Promega,USA) using the heat-shock method [31]. The transformants were selected on low salt Luria-Bertani (LB) agar medium (1% tryptone, 0.5% yeast extract, 0.5% NaCl, and 1.5% agar, pH 7.5) containing ampicillin (100 μg/mL), 0.2 mM,5-bromo-4-chloro-indolyl-D-galactoside (X-gal) and, 40 μg/mL isopropyl-thio-β-D-galactopyranoside (IPTG). Plasmid DNA extraction was carried out on selected white colonies of both genomic and cDNA amplified *BGL*1 clones and then custom sequenced (Macrogen, Korea). The sequence confirmed that recombinant *BGL*I clones were amplified from genomic DNA and cDNA of *T. virens* and were designated as pGEM-T/g*BGL*I and pGEM-T/c*BGL*I respectively. Both recombinant clones were digested with *EcoR*I and *Xba*I restriction enzymes, purified and ligated separately into pGAPZα vector. Ligated products were transformed into *E.coli* JM109 and the transformants were selected in zeocin (25 μg/mL) antibiotic containing low salt LB agar plates. The resulting clones were designated as pGAPZα/g*BGL*I and pGAPZα/c*BGL*I.

### Transformation into *Saccharomyces cerevisiae*

Both pGAPZα/g*BGL*I and pGAPZα/c*BGL*I recombinant vectors were linearized using *Bgl* II and purified. S*accharomyces cerevisiae* (NCYC 87) was inoculated into 0.5 mL YPD broth (1% yeast extract, 2% peptone, 2% glucose) in a 1.5 mL microcentrifuge tube and incubated at 37 °C overnight in a rotary shaker. A volume of 500 μL from the above grown culture was inoculated into 50 mL of YPD broth in a 250 mL conical flask and incubated in a shaking water bath (150 rpm) at 37 °C until the OD_600_ reached 1.4. Yeast electro competent cells were prepared according to the procedure given in the pGAPZα vector manual (Invitrogen, USA). A volume of 80 μL *S.cerevisiae* competent cells was separately mixed with 5–10 μg of linearized pGAPZα/g*BGL*I and pGAPZα/c*BGL*I plasmid DNA. The mixture was subjected to electroporation under optimized conditions, (1.5 Kw, 200 mA and 25 uF and pulse time of 5 ms) in a 0.2 cm electroporation cuvette. The resulting transformation mixture was spread on to YPDS (1% yeast extract, 2% peptone, 2% glucose, 2% agar and 1 M sorbitol) plates with 100 μg/mL zeocin as the selection marker. The plates were incubated for 3 days at 37 °C to obtain positive transformants. Twenty yeast colonies were selected and streaked on fresh YPDS plates containing zeocin (100 μg/mL).

### Screening for recombinant *Saccharomyces cerevisiae*

Colony PCR was performed on the above selected colonies to confirm the presence of the integrated *BGL*I in the *S.cerevisiae* genome. A non-recombinant *S.cerevisiae* and recombinant plasmids (pGAPZα/g*BGL*I and pGAPZα/c*BGL*I) were used as the negative and positive controls respectively. All PCR amplified products were subjected to agarose (0.8%) gel electrophoresis. Two putative clones designated as Y-pGAPZα/g*BGL*I and Y-pGAPZα/c*BGL*I were custom sequenced.

### SDS-PAGE and expression analysis of *BGL*I containing recombinant *S.cerevisiae*

Recombinant *S.cerevisiae,* Y-pGAPZα/g*BGL*I and Y-pGAPZα/c*BGL*I were separately inoculated into YP (1% yeast extract, 2% peptone) medium containing 2% cellobiose broth. Cultures were incubated overnight at 37 °C in a rotary shaker at 200 rpm. The non-recombinant *S.cerevisiae* was used as the control. The enzyme was harvested by centrifugation of the culture medium at 12000 rpm for 2 min at 4 °C. The enzyme extract was concentrated by freeze drying. SDS-PAGE was conducted as described in Sambrook and Russel (2001).

Enzyme activity assay was carried out on the Y-pGAPZα/g*BGL*I and Y-pGAPZα/c*BGL*I recombinant *S.cerevisiae* using the non-recombinant *S.cerevisiae* as the control. The assay was carried out in triplicate with biologically independent clones. They were separately inoculated into 0.5 mL of YPD broth cultures in 1.5 mL microcentrifuge tubes and incubated for 3 days in a rotary shaker at 200 rpm at 37 °C. The enzyme harvests were freeze dried and dissolved in de-ionized water. The enzyme activity of the extracts was quantitatively determined as described above. Both enzyme and substrate controls were maintained throughout the assay procedure.

### Fermentation studies of the recombinant Y-pGAPZα/g*BGL*I and Y-pGAPZα/c*BGL*I

Both Y-pGAPZα/g*BGL*I, Y-pGAPZα/c*BGL*I and non-recombinant *S.cerevisiae* were separately inoculated into YP broth (1% yeast extract, 2% peptone) with 5% cellobiose as the sole carbon source in 100 mL conical flasks. Anaerobic conditions were maintained by nitrogen gas supply at 25 °C at pH 4.5. During fermentation, samples were collected from day 1 to day 7 and centrifuged at 6200 *g* at 4 °C for 10 min to obtain cell free extracts and then it was stored at 4 °C. All experiments were performed in triplicate. The ethanol concentration in the extract was determined by a colourimetric ethanol assay (Megazyme, Ireland) using a standard graph.

### Homology modeling and molecular dynamics simulation studies of BGLI

The tertiary structure of BGLI protein was constructed using the MODELLER (version 9.13) program [32, 33]. The Blast Protein tool [34] was used to search the RSCB PDB protein databank [35, 36] to find X-ray crystallographic structures with sequences similar to the target. Multiple sequence alignment was used for homology modeling and the generated model was based on the beta-glucosidase templates; 3AHY.pdb, 4MDP.pdb, 4MDO.pdb in the RSCB PDB protein databank.

Validity of the model was evaluated using various structure validation tools; VERIFY3D [37] was used to analyze the compatibility of the model with its amino acid sequence, PROCHECK [38] was applied to verify the geometrical and stereo-chemical constraints of the model, the overall quality factor was generated by ERRAT [39]. The binding site of the 3-D model generated above was identified using the COACH server (zhanglab.ccmb.med.umich.edu/COACH) [40, 41].

The 3D structure of the cellulose ligand consisting of five monomer units was constructed and geometrically optimized with 6-31 g** basis set using Gaussian 09 (linux version) software [42]. The optimized ligand structure was docked (Flexible Docking method) into the active site of the model structure of BGLI using DOCK6 software [43, 44]. The grid score energies were used to rank the binding strength of the protein and the ligand [45]. A low score is always a good indicator of high affinity.

The docked complex (protein + ligand) with lowest binding energy obtained from the docking process was selected as the starting configuration for molecular dynamics (MD) simulations using the GROMACS v4.6.5 [46]. The GROMOS54a7 all atom force field was employed for the model protein and the force field parameters for the ligand were obtained from PRODRG server [47]. The protein-ligand complex was placed in the center of a box of 9 × 9 × 9 nm^3^ volume. Na^+^ ions were added to maintain electro neutrality and the system was solvated with SPC/E water molecules [48]. Electrostatic interactions were modeled by particle mesh Ewald (PME) with a short-range cutoff of 1.2 nm [49]. Temperature and pressure of the system were maintained at 300 K and 1 bar using Berendsen’s weak coupling algorithm [50]. All bonds were constrained at their equilibrium distances using LINCS algorithm [51] while other internal motions (bending and torsion) were allowed during molecular dynamics simulation. The system was subjected to 2000 steps of energy minimization with steepest decent algorithm followed by 200 ps long MD simulation to equilibrate the simulation system. Following the equilibration step, 15 ns MD simulation was carried out using a desktop computer with Intel® Core™ i7–950 Processor. Configurations of the system at every 2 ps intervals were stored for further analysis. At the end of the simulation, the non-covalent interactions between ligand and the protein were analyzed by the LigPlot + v.14.5 software [52]. Further, the same simulation protocol mentioned above was employed for 15 ns MD simulation for the model protein alone to study its stability in aqueous medium.

## Results and discussion

**Fig. 1:**
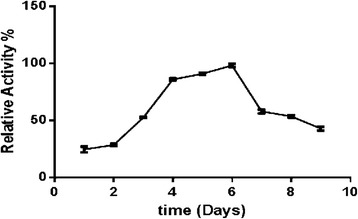
Determination of β-glucosidase I (BGLI) enzyme activity against time of enzyme harvest from Mandel’s medium (MM) containing cellobiose broth cultures at pH optimum (pH 6.5) at 25 °C

The BGLI enzyme activity of *Trichoderma virens* was plotted against the time of broth extraction (Fig. 1). The maximum BGLI activity was determined as 1.5 x 10^−3^ IU ml^−1^ in the culture supernatant on the sixth day. Therefore, isolation of total RNA and the construction of the cDNA were carried out on day six old cultures grown under optimized growth conditions (6.5pH and 25 °C).

PCR amplification of both genomic DNA and cDNA using *BGL*I gene specific primes yielded approximately a 1.4 kb fragment and sequence analysis of their recombinant clones revealed pGEM-T/g*BGLI* to contain a 1439 bp insert while pGEM-T/c*BGLI* contained a 1363 bp insert (GenBank: KU535892.1). The genomic clone (GenBank: KM052276.1) consists of a single intron, 76 bp, (41 bp to 117 bp). Sequence similarity searching (NCBI blast searching and EMBL-EBI similarity searching) indicated that the amplified *BGL*I sequence had 90% similarity to the gene sequence of endo-1,4-beta-glucosidase of *Trichoderma atroviride* (clone JGIBTOG-13A22 (AC237343.1)) and only 83% similarity to *Trichoderma viride.* The similarity of translated open reading frame of the amplified *BGL*I fragment was 99% identical to the amino acid sequence of the endo-1,4-beta-glucosidase of *Trichoderma atroviride*. The InterProScan server protein domain analysis predicted the BGLI catalytic domain to belong to the glycoside hydrolase family 1 and BGLB super family.

**Fig. 2:**
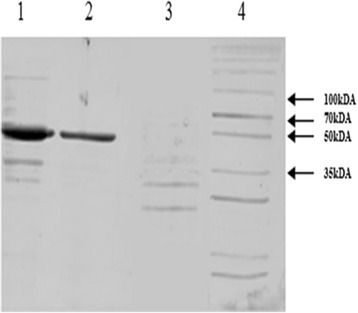
Lanes 1 and 2: BGLI enzyme (52 kDA) secreted by recombinant *S.cerevisiae* clones Y-pGAPZα/g*BGL*I and Y-pGAPZα/c*BGL*I respectively. Lane 3: Enzyme extract of non-recombinant *S.cerevisiae*. Lane 4: Broad range protein molecular weight marker

The theoretical molecular weight of BGLI protein was calculated to be 52 kDa and isoelectric pH was 5.4. A signal peptide sequence was not identified by the signalP-4.1 server. This information shows that the BGL1 is an intracellular protein in *Trichoderma*. Two O -glycosylation sites at positions 306 and 307 and two N- glycosylation sites at positions 55 and 367 of the amino acid sequence were predicted using NetOGlyc 4.0 Server and NetNGlyc 1.0 Server. SDS-PAGE analysis confirmed the BGLI recombinant enzyme expressed by both genomic and cDNA clones to be ~52 kDA (Fig. 2)**.**

The BGLI enzyme activities expressed by Y-pGAPZα/g*BGL*I) and Y-pGAPZα/c*BGL*I *S.cerevisiae* clones were 1.7 x 10^−3^ IU ml^−1^ and 5 x 10^−4^ IU ml^−1^respectively. The BGLI activity observed for pGAPZα/g*BGL*I was comparable to the activity of locally isolated *Trichoderma virens* (1.5 x 10^−3^ IU ml^−1^) denoting the successful approach of genetic engineering in the heterologous extracellular expression of BGLI using the (GAP) promoter driven expression system. Furthermore, the BGLI activity of pGAPZα/g*BGL*I clone was 3.4 times higher than that of the cDNA clone. This represents the direct and/or indirect intron mediated enhancement (IME) of eukaryotic gene expression [53–55]. Increased expression of genes have been observed in recombinant constructs containing an intron compared to its intronless cDNA clones [55, 56]. Present observations support this view. It is possible that the intronic region of the *BGL*I gene is positively influencing the transcription of the gene. The intronic region involved in IME should be within the transcribed region and located close to the start codon of the gene and should be in their natural orientation to increase the expression by enhancing RNA polymerase II initiation and processivity [55]. The intron (41 bp to 117 bp) in Y-pGAPZα/g*BGL*I clone is located following the first exon of the gene. Apart from the above, it has been reported that promoter proximal introns (5′- proximal introns) may be repositories for transcriptional regulatory elements such as enhancers, repressors and elements that modulate the function of the upstream promoter [57–59]. Furthermore, studies on post splicing mechanisms have indicated that the Exon Junction Complex (EJC) consisting of several proteins play a major role in facilitating mRNA metabolism including pre mRNA splicing, mRNA export and association with spliced mRNA in the cytoplasm [60–62]. Ultimately this has been shown to facilitate translation, mRNA localization and protein folding efficiency [62–64].

**Fig. 3:**
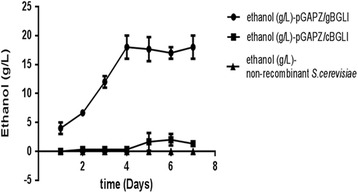
Representation of ethanol produced in the fermented medium containing YP broth (1% yeast extract, 2% peptone) with 5% cellobiose from day 1 to day 7 by recombinant Y-pGAPZα/g*BGL*I and Y-pGAPZα/c*BGL*I against the control of non-recombinant *S.cerevisiae* under anaerobic conditions (pH 4.5 at 25 °C)

The ethanol produced by recombinant Y-pGAPZα/g*BGL*I and Y-pGAPZα/c*BGL*I in the culture supernant of YP broth containing 5% cellobiose against time under anaerobic conditions at 30 °C is represented in Fig. 3. The non-recombinant *S. cerevisiae* was used as the control. The maximum production of ethanol was 18 g/L (0.36 g/1 g of cellobiose) by Y-pGAPZα/g*BGL*I and 2 g/L (0.06 g /1 g of cellobiose) by Y-pGAPZα/c*BGL*I from 5% of cellobiose in 50 ml of culture medium respectively on day four and day five of the fermentation.

Literature cites successful research on expression of β-glucosidase in *S.cerevisiae* from both eukaryotic and prokaryotic sources with the objective of simultaneous saccharification and direct fermentation (SSF) of cellobiose into ethanol [65–67]. Two recent studies report 0.47 g and 0.30 g of ethanol production per 1 g of cellobiose and pretreated cellulose respectively by intracellular expression of fungal BGL in *S.cerevisiae* [65, 68]. In both studies, cellodextrin transporter was introduced together with the *BGL* gene into *S.cerevisiae*. Therefore, the engineered *S.cerevisiae* was able to assimilate cellobiose and cello-oligosaccharides directly into the cell by cellodextrin transporter for intracellular hydrolysis. A similar result (0.36 g ethanol/1 g of cellobiose) was observed in the present study where BGLI expression was under the control of the glyceraldehyde 3-phosphate dehydrogenase (GAP) promoter driven yeast integrative vector (pGAPZα) and α-mating factor driven extracellular secretion. Although it is reported that the above vector has better tolerance to inhibitors, namely furan derivatives, weak and phenolic compounds produced during the anaerobic fermentation of lignocellulosic biomass [69] the ethanol production was stationary after day four in Y-pGAPZα/g*BGL*I fermentation. However, this could also have been due to the limitation of growth factors of *S.cerevisiae*, retention of inhibitory un-hydrolyzed cellobiose and the lethal effect of ethanol on *S.cerevisiae*, thus limiting the enzymatic action of BGLI. One possible means of eliminating the inhibitory effect is by the continuous removal of ethanol from the medium during fermentation and it should be considered in further optimization studies to increase the ethanol yield.

In homology modeling, five probable models obtained from the MODELLER 9.13 software were ranked with respect to their normalized Discrete Optimized Protein Energy (zDOPE) and GA341 score and the model with the best scores was selected as the theoretical model for BGLI protein. This theoretical model contains 455 amino acids, 3654 atoms and 3762 bonds. Characterization of the BGLI with DSSP program [70] indicates that the secondary structure consists of 19 α-helices and 14 β-sheets as given below.

- Alpha helices:
  - (14ALA-17ILE), (23LYS-25GLY), (30ILE-36ALA), (57THR-67LEU), (78TRP-81ILE), (93LYS-109ALA), (124GLU-130TYR), (132GLY-134LEU), (138GLU-153SER), (166PRO-176SER), (188GLU-215SER), (236PRO-258TYR), (264ALA-273ARG), (279ALA-285VAL), (343ALA-357TYR), (379LYS-383LEU), (386ASP-406ASP), (424TRP-429VAL), (449LYS-453SER)
- β-sheets:
  - (7GLN-11ALA), (71SER-75SER), (112THR-116THR), (159ASN-161ILE), (218GLN-220GLY), (222VAL-224ASN), (227PHE-230PRO), (292TYR-295ASN), (299SER-304HIS), (318VAL-321LEU), (362ILE- 366GLU), (410VAL-415ALA), (435THR-438ASP), (444GLN-447PRO)

**Fig. 4:**
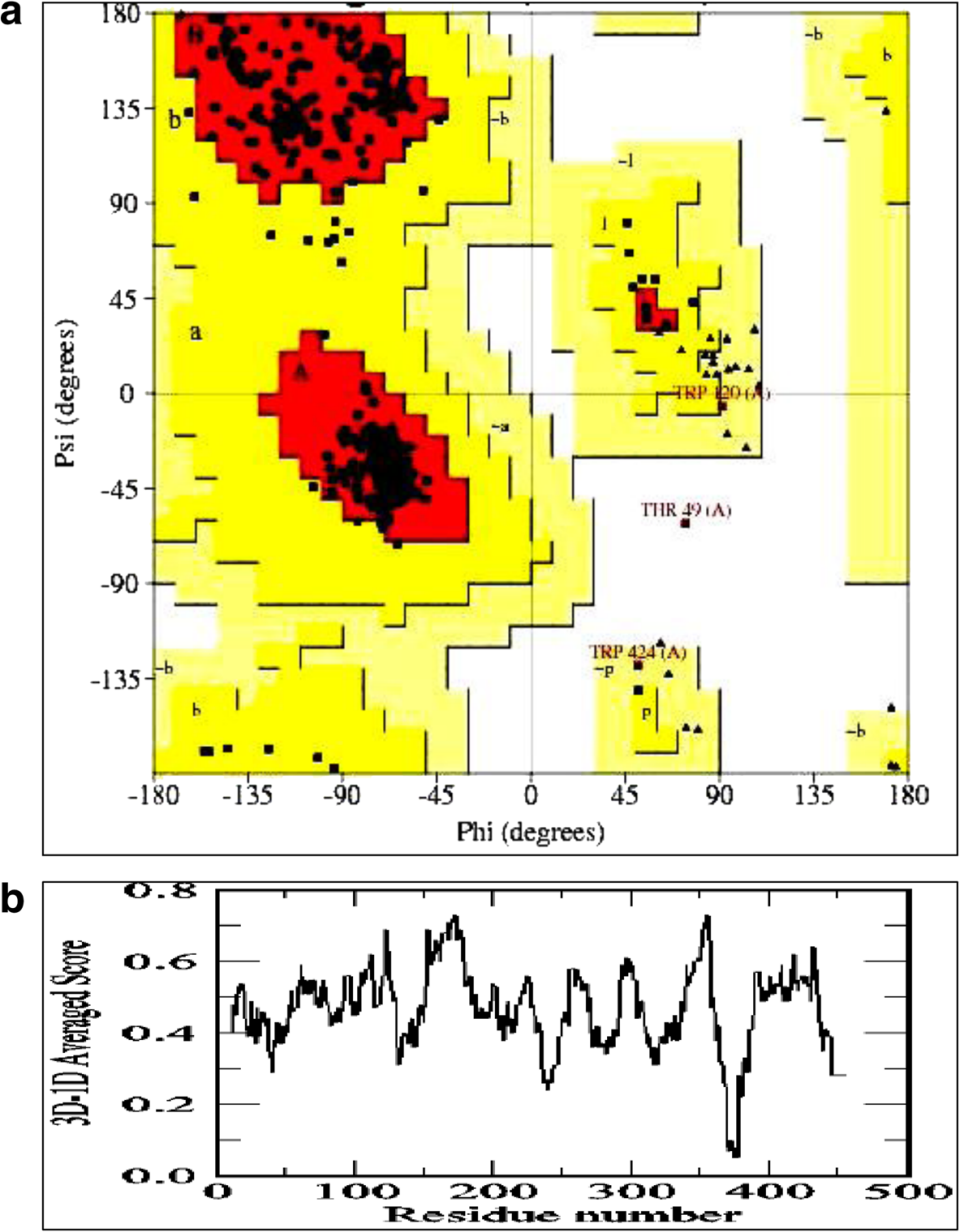
**a** Ramachandran map of modeled BGLI protein. **b** Verify 3D score profile

PROCHECK, VERIFY3D and ERRAT programs were used for the validation of the predicted model. PROCHECK analysis of BGLI is given in Table 1 and the Ramachandran plot generated by the same program is depicted in Fig. 4a. The statistical score of the Ramachandran plot shows that only 0.3% of amino acids are in the disallowed region.

**Table 1:** Statistics of the 3D model of BGLI from the Ramachandran plot

Ramachandran plot statistics	BGLI	
Amino acids in most favored regions	358	91.6%
Amino acids in additional allowed regions	30	7.7%
Amino acids in generously allowed regions	2	0.5%
Amino acids in disallowed regions	1	0.3%
Number of non-glycine and non-proline residues	391	
Number of end residues	2	
Number of glycine residues	37	
Number of proline residues	25	
Total number of residues	455

VERIFY3D profile for BGLI protein shows that all the residues have an averaged 3D-1D score greater than zero (Fig. 4b) while 98.02% residues show more than 0.2 of averaged 3D-1D score. When 80% of residues show an average score of greater than 0.2%, the 3D structure of the protein is considered as reliable [37]. Further, the ERRAT program evaluated the overall quality factor as 88.4 for the modeled 3D structure of BGLI. Therefore considering the above results it can be concluded that the predicted 3D structure of BGLI is highly reliable.

For the molecular docking step, the BGLI-cellobiose complex with the lowest binding energy was selected. It was interesting to note that the selected ligand molecule successfully docked to the binding site which was previously identified by the I-TASSER-COACH Server. Residues; 16GLN, 119HIS, 120TRP, 164ASN, 165GLU, 297TYR, 338TRP, 366GLU, 416TRP, 423GLU, 424TRP, 432PHR are predicted by the COACH server as the consensus binding residues.

**Fig. 5:**
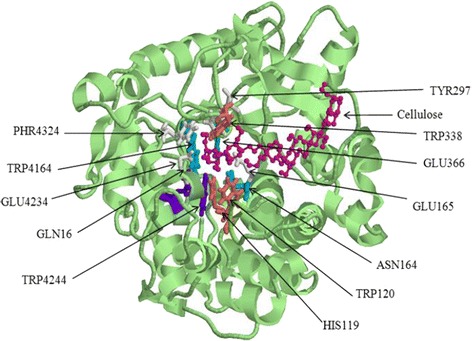
Protein-ligand docked complex

The recorded best grid score for the cellulose-protein was −121.07 kJ/mol and it indicates a fairly high binding affinity value and cellulose bind in a compatible binding pose. The best docked complex is presented in Fig. 5. This value represents the summation of van der Waals dispersive and electrostatic interaction energy, which approximately indicates the binding energy of the ligand.

The general catalytic mechanisms for glycoside hydrolases were proposed decades ago. Most glycoside hydrolases follow either retaining or inverting mechanisms [71]. These reactions typically occur with the assistance of acidic and a basic amino acid residues. The docking results revealed that the presence of glutamic and aspartic acids located in the active site may assist any of the above mechanisms.

**Fig. 6:**
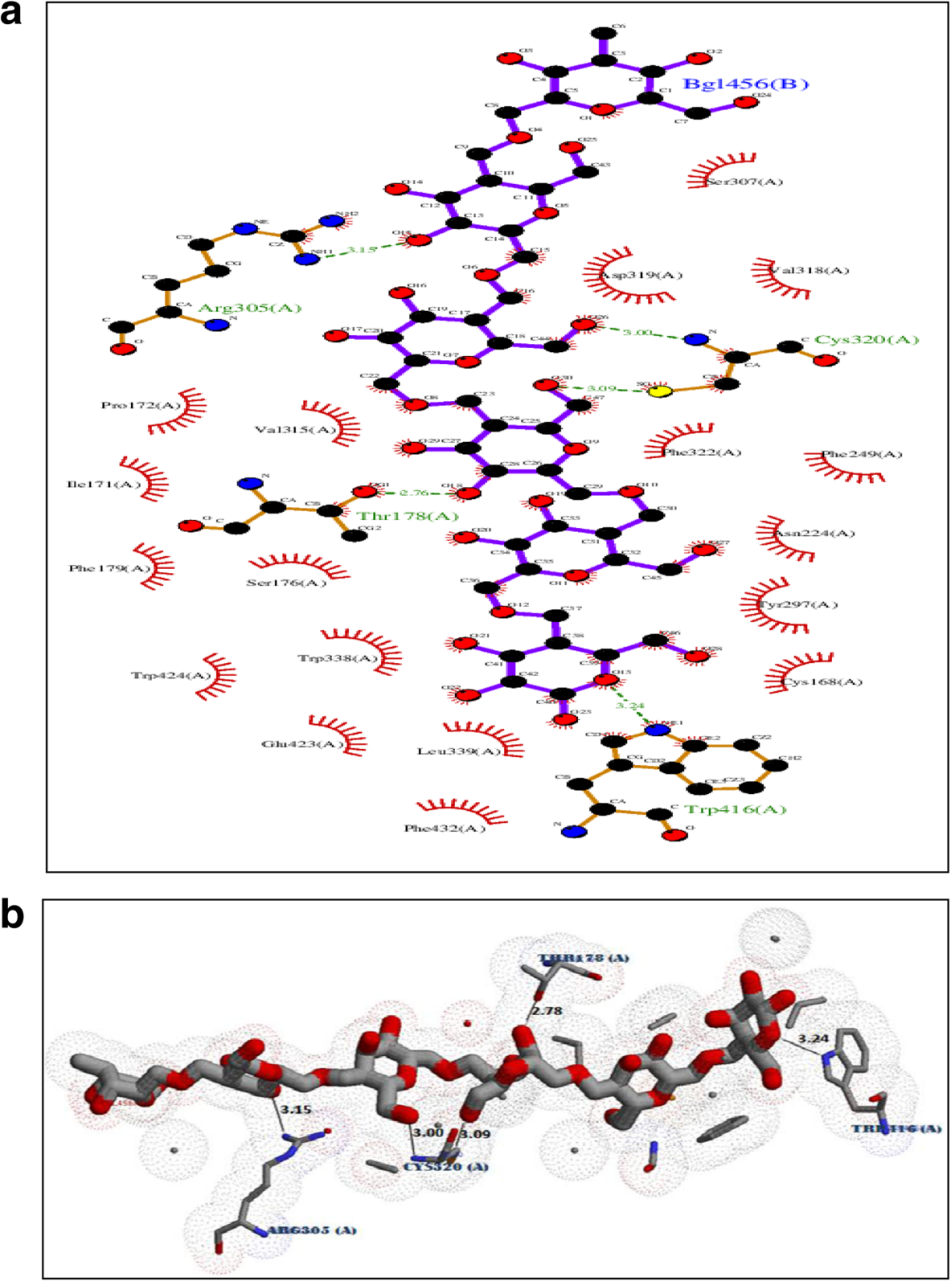
**a** Three dimensional view of H bonds between ligand and the protein residues. **b** H bonds between ligand and the protein residues from LigPlot program

Two MD simulations of 15 ns each were carried out for the protein-ligand complex and the bare protein in aqueous medium with 21,923 SPC water molecules. The non-covalent interaction (H bond) of the final configuration (after 15 ns) of protein-ligand complex identified from LigPlot + v.145 software is presented in Fig. 6a & b. The LigPlot analysis of the protein structure indicates that the ligand forms strong five hydrogen bonds with THR178 (2.76 Å), ARG305 (3.15 Å), CYS320 (3.00 Å) (3.09 Å), TRP416 (3.24 Å). Detailed information is presented in Table 2. The stability of all these H bonds was studied using *g_dist* tool in the GROMACS program.

**Table 2:** Detailed information of H bonds formed between ligand and the protein

Residue	Amino acid	Distance H-A	Distance D-A	Donor Angle	Protein donor	Side chain
178	THR	1.82	2.76	155.04	yes	yes
224	ASN	3.17	3.69	144.24	no	yes
305	ARG	2.58	3.15	116.58	yes	yes
305	ARG	3.33	3.65	100.43	yes	yes
320	CYS	2.08	3.00	152.99	yes	no
320	CYS	2.39	3.10	126.54	no	no
416	TRP	3.67	3.24	116.59	yes	yes

The analysis indicates that the distance between the centers of mass of the two groups of atoms which formed H bonds was nearly constant during the total simulation time. These results reveal the stability and effectiveness of the H bonding.

**Fig. 7:**
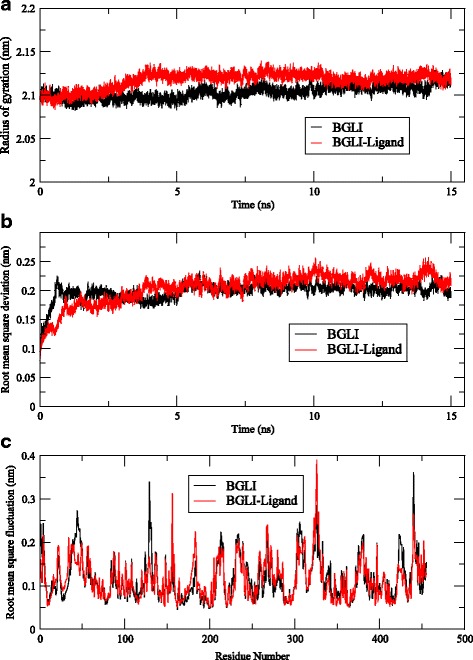
**a** Root mean square deviations (RMSD) of the backbone. **b** radius of gyration (Rg) of the protein from 15 ns long MD trajectory. **c** Root mean square fluctuation (RMSF) of the residues in the protein over 15 ns long MD trajectory

The stability of the protein after forming a complex with cellulose was studied by calculating root mean square deviations (RMSD), radius of gyration (Rg) and root mean square fluctuation (RMSF) of the protein. All three parameters of the protein of the complex were compared with that of the bare protein. Figure 7a & b compares RMSD of the backbone of the protein in two systems. Both systems indicate stable structures with RMSD of about 0.2 nm and there is no indication of increasing the RMSDs with time. Figure 7b gives the variation of radius of gyration (Rg) as a function of simulation time which indicates the compactness of the protein. As seen in the Fig. 7b, Rg of both systems were maintained approximately at the same value. Both these results suggest that the BGLI preserves its tertiary structure even after making a complex with the ligand.

Figure 7c represents the root mean square fluctuation (RMSF) of the protein residues in both simulation systems (protein alone and the protein with the substrate) indicating stable 3D structures for the bare protein and the protein in the complex. It is observed that most of the fluctuations are concentrated in the region of residues 323 and 328, residues 156 and 440 for both systems. Residues 44, 129–131 show relatively higher fluctuations in the free protein. Further, none of the high fluctuating residues of the protein of the complex were in the predicted active site and it can be safely postulated that the enzyme can perform well with the bound ligand via the proposed two mechanisms.

## Conclusion

The genomic and cDNA of β-glucosidase 1 (BGL1) were isolated from *Trichoderma virens* and successfully characterized, cloned and expressed in *S.cerevisiae*. The expression of *S.cerevisiae* genomic DNA clone was determined to be higher than its cDNA clone. In the fermentation study a higher amount of ethanol (0.36 g/1 g of cellobiose) was obtained by *S. cerevisiae* genomic DNA clone than its cDNA (0.06 g/1 g of cellobiose). BGLI carrying *S.cerevisiae* will have the potential to be used in the industrial production of ethanol by the hydrolysis of the cellulose component in plant biomass by the combinatory simultaneous actions of endoglucanase and cellobiohydrolase, the other two enzymes of the cellulase complex.

The major ligand binding domain of the model enzyme was identified from the results of molecular docking studies. MD simulation results indicate an overall stable confirmation of BGL-cellobiose complex that exhibits an almost similar structural flexibility shown by the free enzyme. Further, it has been found that mainly five hydrogen bonds are involved in maintaining the enzyme-substrate association. Thus these results lead to clear understanding of its binding site. The predicted model was a realistic stable model and the predicted active site residues would be a good starting point for the further efforts in the rational design of mutagenic experiments aimed at improving the catalytic activity of glycoside hydrolases.
